# Functional connectivity of the striatum in experts of stenography

**DOI:** 10.1002/brb3.333

**Published:** 2015-03-25

**Authors:** Takehito Ito, Tetsuya Matsuda, Shinsuke Shimojo

**Affiliations:** 1 Brain Science Institute, Tamagawa University6-1-1 Tamagawa Gakuen, Machida, Tokyo, 194-8610, Japan; 2 Molecular Neuroimaging Program, Molecular Imaging Center, National Institute of Radiological Sciences4-9-1 Anagawa, Inage-ku, Chiba-shi, Chiba, 263-8555, Japan; 3 Division of Biology and Biological Engineering/Computation and Neural Systems, California Institute of Technology139-74, Pasadena, California, 91125

**Keywords:** Long-term neural plasticity, putamen, stenography

## Abstract

**Introduction:**

Stenography, or shorthand, is a unique set of skills that involves intensive training which is nearly life-long and orchestrating various brain functional modules, including auditory, linguistic, cognitive, mnemonic, and motor. Stenography provides cognitive neuroscientists with a unique opportunity to investigate the neural mechanisms underlying the neural plasticity that enables such a high degree of expertise. However, shorthand is quickly being replaced with voice recognition technology. We took this nearly final opportunity to scan the brains of the last alive shorthand experts of the Japanese language.

**Methods:**

Thirteen right-handed stenographers and fourteen right-handed controls participated in the functional magnetic resonance imaging (fMRI) study.

**Results:**

The fMRI data revealed plastic reorganization of the neural circuits around the putamen. The acquisition of expert skills was accompanied by structural and functional changes in the area. The posterior putamen is known as the execution center of acquired sensorimotor skills. Compared to nonexperts, the posterior putamen in stenographers had high covariation with the cerebellum and midbrain.The stenographers' brain developed different neural circuits from those of the nonexpert brain.

**Conclusions:**

The current data illustrate the vigorous plasticity in the putamen and in its connectivity to other relevant areas in the expert brain. This is a case of vigorous neural plastic reorganization in response to massive overtraining, which is rare especially considering that it occurred in adulthood.

## Introduction

Stenography, or shorthand, is a medium that is used to bridge regular spoken and written language. It consists of a unique set of skills that involves various critical brain modules, including auditory, linguistic, cognitive, mnemonic, and motor modules. Until recently, professionals from different countries or a language background have used stenography to record significant speeches and meetings, including the Diet records.

**Figure 1:**
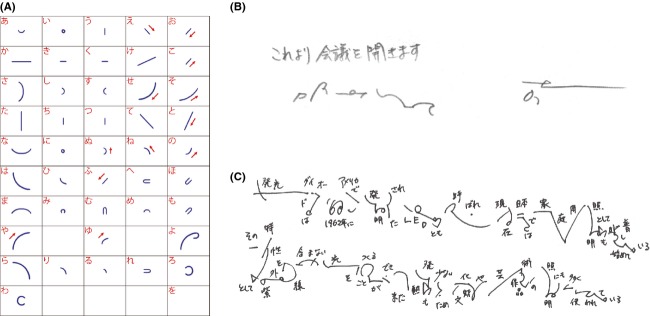
Correspondence between the original text and the stenography. At first, all trainees learnt the basic stenographic characters as shown in (A), which was the Japanese Kana syllabi, but the top-notch stenographers used the their own original symbols to cut down the dictation amount and wrote down more rapidly and effectively, like (B) and (C). (B) The Japanese text (upper left) stating “Now, start a meeting,” the left lower stenography was written by the Kana syllabi of the stenography, and the right lower stenography was extremely shortened by the top-notch stenographer. (C) Examples of Japanese texts written in stenography and mapping onto the regular Japanese writing. As one can see, the original texts were shortened drastically by the stenographic symbols.

The top-level experts in shorthand of the Japanese language can listen to and write down 340–400 letters/min, which is approximately 5–6 times faster than what nonexperts can do. Shorthand in Japan originated in the 1880s when a few different handwriting systems were developed, most of which were based on simplified syllabic symbols (unlike Hindi/European languages in which stenography is mainly based on phonetics). Compared to other natural languages, Japanese shorthand is especially challenging because of the complex letter types in Japanese (hiragana, katakana, kanji, etc.; Fig.1) and other factors including vocabulary, flexibility in word order, redundancy, and verbalization ambiguity.

Shorthand is a distinctive type of expertise because it requires long-term intensive learning and because training is received mostly in adulthood. It raises the intriguing question about how much of their ability can be explained by neural plasticity in the anatomical and functional sense? Very few reports on this subject are available in the literature (Chang 2014). Unfortunately, such extreme stenographers' brain are currently disappearing, with the Japanese national Diet record being one of the last places where shorthand was replaced with a voice recognition system in March 2011. In the vast majority of Western countries, shorthand was replaced with voice recognition systems years earlier. Although there are 810 (semi)professional members currently registered in the Japanese Association of Stenography, only 70 of them who are engaged in the National Diet Record were considered to be the best. We used fMRI to scan the brains of these professionals in order to obtain insights into how the brain can plastically reorganize itself under such extreme pressures on accuracy and speed through a lifelong training regimen that begins mostly in adulthood.

Expertise in stenography is unique in various aspects compared to other proficiencies in perceptual and motor skills. Stenographers have been intensively trained over many years in their adolescence and adulthood. They use these skills routinely and have high professional motivation to maintain and improve their skills. Expertise in stenography itself requires the recruitment of various sorts of skills and neural circuits, as described later. Each of these functional modules or circuits (and their combinations) offers a hypothesis for the neural correlates of stenographic expertise. In addition, there is the possibility that their expert performance is based on reactivation of the circuit used for stenography training-specific learning, such as motor learning, which is associated with auditory input (Doyon et al. 2003; Butler et al. 2011; Park et al. 2011; Voss et al. 2011). It is also worth noting that a recent report on top-professional shogi (Japanese chess) players has shown that they exhibit significant activation in the caudate (Wan et al. 2011). The caudate is a part of the striatum that is located in the basal ganglia and that is known as a center of motivational reward (Delgado et al. 2004) and reward-based learning (Haruno et al. 2004). The basal ganglia in the top-professional shogi brain may be responsible for predictions based on past experience and the current game situation. This area is known to have a central role in learning and in executing acquired sequential movements (Jueptner et al. 1997; Floyer-Lea and Matthews 2004; Lehéricy et al. 2005; Poldrack et al. 2005; Jankowski et al. 2009; Reithler et al. 2010; Steele and Penhune 2010). This makes it a strong candidate region for the control or integration of multimodal functions in the brain of stenographers. To investigate these possibilities, we scanned samples of expert shorthanded brains to determine the impact of professional training on functional representation with the multimodal functional components in stenography, by comparing writing, imagery, and hearing conditions. (This design is meant to isolate the neural correlates of functional coordination for the stenograph task per se, as opposed to elementary components such as auditory and motor; later.)

**Figure 2:**
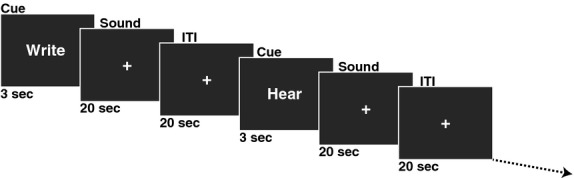
Task design. The functional magnetic resonance imaging (fMRI) paradigm consisted of the following five conditions: writing, air-writing, imagining, hearing, and reverse-sound. Because of technical failure, we excluded the air-writing condition in the following fMRI data analysis. Each condition appeared four times in random order in one scan. The duration of each condition was 20 sec, and the interstimuli interval (ITI) was 20 sec. In the writing condition, subjects were asked to write down the speech itself, which was coming from the headphones, with a plastic pen on an acrylic writing table. The participants could see their own hand and the writing table through combined mirrors. In the Imagining condition, they imaged the writing motion without actually moving. In the hearing condition, they only heard the speech. In the reversed sound condition, they only heard the reversed audio speech.

Because of the complexity of Japanese letters (hiragana, katakana, and kanji) and their grammatical structure and intrinsic ambiguity, Japanese stenography is extremely complex and more demanding than other languages. Therefore, we expected extensive unique mobilization of multiple modules/circuits in Japanese stenographers compared with individuals with other proficiencies. From a neural network perspective, stenographers would need to develop multiple cognitive or neural bases that are mainly located in dictation-related regions. These include regions involved in (1) the automatic linguistic processing of vocal sounds: the temporal gyrus (Woods and Alain 2009), and the supramarginal gyrus (Gow 2012); (2) the automatic control of hand movements: the precentral gyrus (Carnell et al. 2012), the postcentral gyrus (Ma et al. 2011), the lateral and the medial premotor cortex, the cerebellum, and the putamen (Jueptner et al. 1997; Floyer-Lea and Matthews 2004; Lehéricy et al. 2005; Poldrack et al. 2005; Jankowski et al. 2009; Reithler et al. 2010; Steele and Penhune 2010); and (3) the ability to concentrate attention and to cope with multiple tasks, including predictions under severe time pressures: the inferior parietal cortex, the supplementary motor area, and the dorsolateral prefrontal cortex (Numminen et al. 2004). Moreover, several neural circuits or networks might also need to be plastically reorganized, and examples of these include the cortical-striatal-cerebellar network (Mosier et al. 2011), the parietofrontal network (Andersen and Cui 2009), the sensorimotor network (Petroni et al. 2010), and the language-processing network (Choudhury and Mukherjee 2009). Indeed, various kinds of expertise, such as sensorimotor (Dick et al. 2011), athletic (Kim et al. 2008; Wei and Luo 2010), artistic (Herdener et al. 2010; Kleber et al. 2010), cognitive (Western chess (Campitelli et al. 2005), Japanese chess (top-professional shogi) (Wan et al. 2011), creative writing (Shah et al. 2013; Lotze et al. 2014)), and perceptual (Gauthier et al. 2010) are known to reorganize these networks, but very little has been understood specifically on the neural plastic changes due to the stenography training. In addition, because previous experiments have shown that some types of expertise result in morphological changes during lifelong training or work (Maguire et al. 2000; Gaser and Schlaug 2003; Jacini et al. 2009; Jäncke et al. 2009; Park et al. 2011; Di Paola et al. 2013), the stenographers' brain might also exhibit morphological changes in some functional domains. The Japanese language is unique, especially in the aspects described above. Thus, we expected that the lifelong training of stenographers would result in modulated or reorganized multimodal functions or neural networks in the stenographers' brain. To examine the plastic reorganization of the stenographers' brain, we designed a multiple-work task (Fig.2) that required the participants to use the multimodal functional network in several different conditions.

## Materials and Methods

### Participants

Eighteen right-handed stenographers and 23 right-handed controls participated in this study. After checking the translation, data sets with head movements greater than 2 mm were excluded according to standard procedures. The data for 13 right-handed stenographers (10 females; mean ± standard error of the mean (SEM) age, 33.1 ± 1.87; age range, 26–42), and 14 right-handed controls (8 females; 31.1 ± 1.30; 23–38) were analyzed as described. All participants provided written informed consent, which was approved by the ethics committee of Tamagawa University. None of the participants had a history of neurological disorders. All of the stenographers belonged to the branch of the House of Representatives and the House of Councillors in the Japanese Shorthand Association. All stenographers had first-class shorthand skills, and routinely performed stenographic jobs (the average ± standard deviation (SD) for job history was 14.6 ± 6.52 years). A first-grade certification of stenography is required to work as a first-class stenographer in Japan, and, to obtain the first grade, stenographers typically practice stenographic skills at a training school for about 2.5–3 years. Then, one has to pass the first-class examination, which requires an accuracy of more than 98% to be able to write speech with an extraordinarily fast speed, which is faster than the speech of a news announcer. The average ± SD age for the professional stenographers who passed the test was 19.5 ± 1.13 years. None of the controls had received any special shorthand training in their past life.

### Task design

All of the participants underwent the functional magnetic resonance imaging (fMRI) paradigm twice. The fMRI paradigm consisted of the following five conditions (Fig.2): writing, air-writing, imagining, hearing, and reversed-sound conditions. This is meant to compare, and thus to isolate the neural activity/connectivity critically responsible for the stenography task per se, which requires a massive coordination of various functional components, as opposed to each sensory or motor components, such as auditory (both semantic and nonsemantic), motor, mental imagery, etc. Because of a technical failure, we excluded the air-writing condition in the subsequent fMRI data analysis. Each condition appeared four times in random order in one scan. The duration of each condition was 20 sec, and the interstimuli interval was 20 sec. In the writing condition, the participants were asked to write down the speech, which they heard through headphones, with a plastic pen on 16.7 cm × 24.2 cm writing paper on an acrylic writing table. The stenographers wrote the speech using stenography in the writing condition, and the controls wrote the speech in the standard way (i.e., using a mixture of Kanji and Kana). The participants could see their own hand and the writing table through combined mirrors. To prevent head motion, the back of the neck was stabilized with an air-bag cushion, and the head was immobilized with a forehead band. In the Imagining condition, the participants imagined the writing motion without their hand moving. In the hearing condition, the participants only heard the speech; and in the reversed-sound condition, the participants only heard the reversed audio of the speech. The participants were asked not to move their hands and arms during Imagining, and we interviewed them later to ascertain their compliance with the instructions. After the fMRI scan, the experimenter examined the dictation accuracy in the writing condition and calculated the correct ratio of each participant as described below. The numbers of syllabic sounds were adjusted between the stenographers and the control subjects to match the task difficulty as addressed in the correct ratio. According to our pilot experiment, the stenographers could write 140–180 syllabic sounds per 20 sec, while the controls wrote 25–35 syllabic sounds per 20 sec. To match the correct ratio of each group, the average number of syllabic sounds in each part of the speech was 163.63 ± 1.12 (SEM) sounds per 20 sec for the stenographers and 31.25 ± 0.484 (SEM) sounds for the controls. Before the trials for the five conditions, all of the subjects practiced writing in a magnetic resonance scanner for 10 min.

### Audio data

The audio data (the speech stimuli) were recorded from a television news analysis program and customized the number of syllabic sounds and the length of the speech.

### Correct ratio

As described above, because of the differences in dictation skills between the two groups, we adjusted the amount of speech to equalize the dictation accuracy in the writing condition between the groups at a 95% accuracy. The average of the syllabic sounds of each condition was 163.6 ± 1.12 (SEM) sounds per 20 sec for the stenographers and 31.2 ± 0.484 (SEM) sounds per 20 sec for the controls. After the fMRI scan, the experimenters and participants compared the written texts with the original texts to confirm the accuracy, counted the number of spelling mistakes and lack of text, and calculated the correct ratio. The average correct ratio of the stenographers was 95.0 ± 0.85% (SEM) and that of the controls was 93.5 ± 0.87% (SEM). There were no statistical differences between the two groups in the correct ratio.

### Hand movement

Because of the differences in the speech speed and the writing systems (stenography vs. regular Kanji/Hiragana) between the two groups, there were also differences in hand movement trajectory and speed. We examined the trajectories in the written text after the scans. In particular, we quantified the total stroke length in terms of the number of pixels. The average number of pixels in each group was 40,301 ± 7734 in the stenographers and 29,787 ± 8508 in the controls. There was a statistical difference (*P *<* *0.001) between the two groups. The stenographers abbreviated their writing frequently, yet they exhibited more hand movement, partly because they dictated much of the squeezed speech and partly because they tended to generate strokes with a larger spatial scale.

### fMRI procedure

The functional imaging was conducted on a 3-TeslaTRIO MRI scanner (Siemens medical solutions, Erlangen, Germany). For each participant, we acquired whole-brain T1-weighted anatomical scans and gradient echo T2*-weighted echo planar images (EPI) with blood oxygen level-dependent contrast (repetition time, 2500 msec; echo time, 25 msec; slice gap, 0.6 mm; field of view, 192 mm; slice thickness, 3.0 mm; 40 oblique axial slices). We used a tilted acquisition sequence at 30° to the anterior commissure-posterior commissure line to recover signal loss in the medial orbitofrontal cortex (Deichmann et al. 2003). In addition, we used a 12-channel head coil for the EPI sequence and a 32-channel head coil for the T1-weighted anatomical scans. Each brain volume comprised 40 axial slices of 3-mm thickness and 3-mm in-plane resolution. Each scan lasted about 25–30 min independent of performance, and the first five volumes of the images were discarded to allow for equilibration effects.

### Imaging data analysis

The imaging data analysis was performed with Statistical Parametric Mapping (SPM) (SPM8; Wellcome Department of Imaging Neuroscience, Institute of Neurology, London, U.K.). To correct for participants' motion, the images were realigned to the mean image. The structural T1 images were coregistered to the mean functional echo planar images for each participant and normalized to a bias-corrected T1-weight image of each subject. Spatial smoothing was applied with a Gaussian kernel with a full width at half maximum of 8 mm.

At the first level of analysis, the statistical models for each participant were computed, and the following three contrasts were calculated for each subject: writing versus reversed, imagining versus reversed, and hearing versus reversed. Finally, the realignment parameters were applied to the image data analysis. Each contrast image was used for the second group analysis of Stenographers, Controls, and Stenographers versus Controls. Each region of interest (ROI) was analyzed with a MarsBar tool for SPM (http://marsbar.sourceforge.net/). For extracting ROI data, we used a spherical (4 mm) ROI for each analysis. All ROI data analyzed with SPSS (IBM Corporation, Armonk, NY) for the group comparison and the correlation analysis with age correction.

### Voxel-based morphometry

T1-weighted image registration was achieved with a diffeomorphic registration algorithm that is implemented in the Diffeomorphic Anatomical Registration Through Exponentiated Lie Algebra (DARTEL) toolbox for SPM8. First, T1-weighted images were classified into gray matter, white matter, and cerebrospinal fluid with a segmentation routine. The resulting parameter files were imported into the DARTEL procedure to produce rigidly aligned gray and white matter tissue classes that were resliced to a 1.5 × 1.5 × 1.5-mm voxel size. We then used the rigidly aligned tissue class images to estimate the nonlinear deformations to best align all of the images. During this estimation stage, DARTEL iterates between building a template and registering tissue class images with the template. We used the resulting flow fields to wrap gray and white matter images for each participant. The spatially normalized images were rescaled by the Jacobian determinants of the deformations by using 64 time points to solve the partial differential equations. To obtain meaningful coordinates of the volume alterations, the final DARTEL template was normalized to the Montreal Neurological Institute space, and the resulting deformations were applied to the gray matter images of each participant with a MATLAB script. Finally, the images were smoothed with an 8 × 8 × 8-mm Gaussian kernel. The input features for the subsequent analysis were smoothed, modulated, and normalized gray matter images. The gray matter group comparisons were modeled with an SPM8 factorial design and corrected for age.

### Psychophysiological interactions

**Figure 3:**
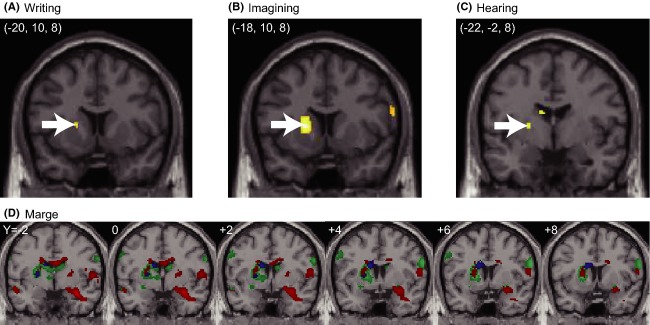
The left putamen exhibited activation in stenographers. A direct group comparison revealed that the stenographers showed higher activation around the anterior putamen compared with the controls. (A–C) A two-sample *t*-test comparison of the stenographers versus the controls revealed that the left putamen of the stenographers exhibited higher activation than that of the controls in all three conditions: writing (A; −20, 10, 8; *P *=* *0.0032), imagining (B; −18, 10, 8; *P *<* *0.001), and hearing (C; −22, −2, 8; *P *=* *0.0019). The white arrows indicate the peak voxels. To define the activated regions, a statistical threshold of *P *<* *0.005 (uncorrected) was used. D) Each contrast showed the stenographers versus the controls in three conditions. The activated regions of writing (red), imagining (green) and hearing (blue) were overlapping with each other. The anterior putamen has a central function of the stenographic brain. To define the overlapping regions, the statistical threshold was *P *<* *0.01 (uncorrected) for each contrast.

To conduct psychophysiological interactions (PPI) (Friston et al. 1997), we extracted the deconvolved time course from the anterior and the posterior putamen for each participant. To create the PPI term, we calculated the product of the deconvolved activation time course and the vector of the psychological variable of interest (1 for writing; −1 for reversed sound). Individual-level PPIs were computed for each participant and then entered into a random-effects group-level regression analysis. The centers of each voxel of interest and ROI were defined by two-sample *t*-tests that compared the stenographers and the controls (Fig.3, Supplementary Fig. S1).

### Statistical analysis

All statistical analyses were performed with SPSS software (IBM Corp. Released 2010. IBM SPSS Statistics for Windows, Version 19.0. Armonk, NY: IBM Corp.).

## Results

### Behavioral Data

As described in the introduction, we adjusted the number of syllabic sounds and the dictation accuracy in the writing condition to a 95% accuracy to push toward the upper limit of the cognitive/motor abilities in both groups. As a result of this adjustment, the average syllabic sounds that the participants could dictate were 163.6 ± 1.12 (SEM) sounds per 20 sec for the stenographers and 31.2 ± 0.484 (SEM) sounds per 20 sec for the controls. The actual percentage correct of the stenographers and the controls in the writing condition was 95.0 ± 0.85% (SEM) and 93.5 ± 0.87% (SEM), respectively. There were no statistical differences between the two groups in the percentages correct.

### The left putamen had a specific function in the stenographers

**Figure 4:**
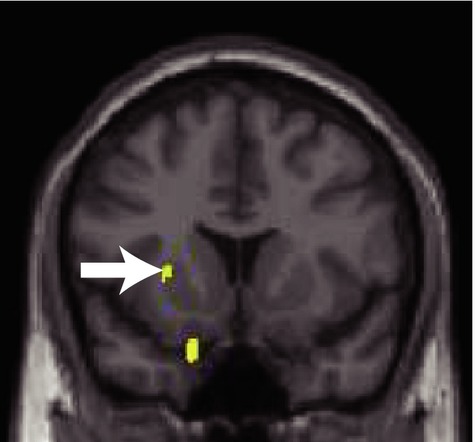
Portions of the left putamen where voxel-based morphometry showed a statistical difference between the stenographers and the controls. The stenographers exhibited an enlarged putamen compared to the controls in the left dorsal anterior part of the putamen. The black arrow indicates the peak voxel (*P *=* *0.0021 with a coordinate of (−24, 14, 5)). To define the activated regions, a statistical threshold of *P *<* *0.005 (uncorrected) was used.

In the writing condition, both groups exhibited high activity in many brain regions, some of which are known to be important for motor, auditory, and memory functions. A group comparison of the fMRI data showed that the left anterior putamen of the stenographers exhibited higher activation than the controls in all conditions (writing, imagining, and hearing; Fig.3A–C, Supplementary Fig. S1A–C), and there were statistical differences regardless of the ROI radii (Supplementary Fig. S1A–C). These regions with higher activation overlapped among the three conditions (Fig.3D) and were localized in the anterior putamen. Morphologically, the left dorsal anterior part of the putamen in the stenographers was enlarged compared to that of the controls (−24, 14, 5; *P *=* *0.0021, Fig.4). This enlarged region overlapped with the region of higher activation.

### Functional modification of the stenographers' left putamen

**Figure 5:**
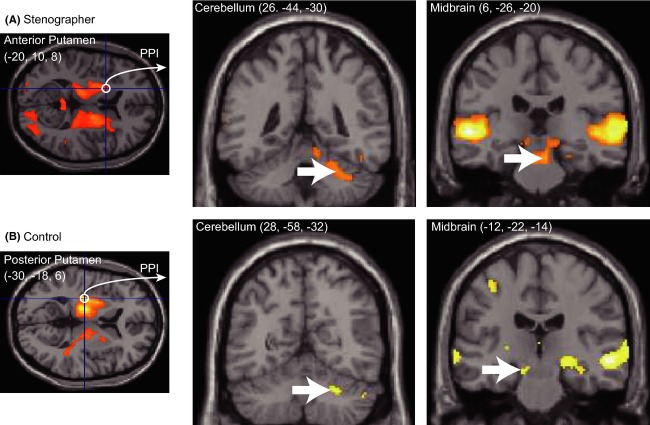
The functional modification of the left putamen of the stenographers. A psychophysiological interaction (PPI) analysis revealed that the regions that covaried with the anterior or posterior putamen were different between the stenographers (A) and the controls (B). The left panels of (A) and (B) show the peak voxels (white circles) in the anterior and posterior putamen, and these were used as the seed regions for the PPI analysis. An uncorrected threshold of *P *<* *0.0001 was applied to the left panels of (A) and (B) and *P *<* *0.001 (uncorrected) was applied to the middle and right panels of (A) and (B). The white arrows indicate the peak voxels.

According to the literature (Jueptner et al. 1997; Lehéricy et al. 2005; Jankowski et al. 2009), the anterior and posterior regions of the putamen have different roles in regulating motor skills. The anterior regions are implicated in the acquisition of new motor skills, whereas the posterior regions are critical for automated skill movements. To examine the functional differences in the anterior putamen and the posterior putamen between the two groups, we performed the PPI analysis (Fig.5), which is a widely accepted technique to estimate the functional connectivity between brain regions (Friston et al. 1997). The anterior putamen (−20, 10, 8) of the stenographers showed a significant covariation with the right cerebellum (26, −44, −30, Fig.5A middle), and the midbrain (6, −26, −20, Fig.5A right). On the other hand, the posterior (−30, −18, 6)*,* but not the anterior (−20, −10, −8) putamen of the controls exhibited significant covariation with the right cerebellum (28, −58, −32, Fig.5B middle) and the midbrain (−12, −22, −14, Fig.5B right). Thus, in general, the putamen of each group had high covariation with the cerebellum and the pons, but a closer look revealed that there were different regions activated between the groups (Fig.5). The PPI analysis showed that the anterior putamen of the stenographers had a region with higher covariation with the midbrain (4, −24, −22 (Supplementary Fig. S2A)) compared to the controls. On the other hand, there were no statistical differences between the two groups around the cerebellum and midbrain with the anterior putamen as the seed region (Supplementary Fig. S2). As shown in Supplementary Fig S3, the posterior putamen of the stenographers and the anterior putamen of the controls did not exhibit any regions of covariation around the cerebellum and midbrain. As an alternative way to address the same issue of functional connectivity, we conducted a PPI analysis with the coordinate (−24, 14, 5), which was the morphologically enlarged region in the left anterior putamen as a seed, and found a region of covariation only around the midbrain (data not shown).

### Correlations of brain activity and job history

Because stenographers use their skills on a regular basis, the length of their career might affect the stenographers' brain functions. To examine this possibility, we extracted the ß value from the brain regions detected in the current study and calculated the correlations between their career, which was defined as the length of their career as a professional stenographer, and the brain activities. Only the activity of the supramarginal gyrus exhibited a negative correlation with a career for the stenographers (Pearson's r, −0.556; *P *=* *0.048) and not for the controls (r, 0.038; *P *=* *0.896), but it may be interpreted as an indirect, emergent outcome of different functional networking between the two groups, as mentioned above. No other area showed activity that correlated with the career.

## Discussion

In the current study, we observed distinctive behavioral characteristics of expert professional stenographers as well as possible neural correlates of them. We demonstrated that the stenographer's putamen has an extremely localized functional core of the stenographic skill. The putamen of stenographers but not that of nonexperts had a high covariation with the cerebellum and midbrain. Moreover, the ventral regions of the putamen were morphologically enlarged. This region overlapped with the region exhibiting a high covariation with the cerebellum. While it is very difficult to draw definitive conclusions because this is the first fMRI study of expert Japanese stenographers and virtually no directly comparable studies are available, we can speculate on how these neural changes contribute to the high performance levels of professional stenographers. The current data suggest that functional convergence centered around the putamen was the characteristic feature of the stenographers' brain, and this enabled the stenographers to execute their high-level performance. Though the current data provided the feasible evidence of the plastic reorganization in the putamen and the relevant areas, our design does not allow us to reveal the neural reorganization within a single professional group (i.e., it was not a pre vs. post comparison). Thus, our interpretation remains to be speculative.

### Functional role of the posterior putamen

Because the posterior (sensorimotor) putamen plays an important role in the execution of automated over learned sequential movements (Jueptner et al. 1997; Floyer-Lea and Matthews 2004; Lehéricy et al. 2005; Poldrack et al. 2005; Jankowski et al. 2009; Reithler et al. 2010; Steele and Penhune 2010), we expected that the posterior putamen of stenographers would be more activated or more effectively functionally connected with other relevant functional modules compared to nonexperts. However, this was not consistent with the current data indicating the anterior, instead of the posterior region in the putamen both functionally and structurally. This inconsistency might be explained by the following factors. First, like the skill-learning fMRI studies described above (Jueptner et al. 1997; Floyer-Lea and Matthews 2004; Lehéricy et al. 2005; Poldrack et al. 2005; Jankowski et al. 2009; Reithler et al. 2010; Steele and Penhune 2010), the training term was a few days or weeks (with the cumulative training time ranging from several hours to several tens of hours), which is in strong contrast to the case of professional stenographers who were intensively trained over years. Second, the vast majority of previous studies on the brain of experts (e.g., sports, music, and games) (Chang 2014) have been limited to the use of relatively simple perceptual or cognitive skills, with little attention paid to more complicated real-world sensorimotor functions. Third, although the anterior putamen has been considered the center of motor acquisition in general, there have been some indications that activation of the anterior putamen is related to motor programming (Alexander and Crutcher 1990; Kimura 1990; Lewis et al. 2004; Haruno and Kawato 2006; Kraft et al. 2007; Aramaki et al. 2010) or more general functions, such as attention (Romo et al. 1992) or intention (Scholz and Kelso 1990; De Luca et al. 2010).

### Automatic vs. executive control

In the current study, the anterior putamen in the stenographers may either operate as a switch between automatic versus attentive processes, or act as the center of integration or connectivity of various functional modules, which can become automatic through intensive training. Indeed, the increase in connectivity with motor-related regions, such as the cerebellum, supplementary motor area (precentral gyrus), and putamen, is known to be important for shifting from the executive stage to the automatic stage (Wu et al. 2008). Thus, the modulated networks of the stenographers' brain imply the involvement of the automatic stage of motor function. It is conceivable that the anterior putamen is activated for other critical multimodal functions of expertise, although no direct report of this has been published. It may be worth noting that not only the stenographers but also the controls had extensively used their writing skills for nearly a lifetime. It was therefore no surprise that nonexperts (the controls) also showed significant activation in the automation-related (posterior) region of the putamen and exhibited a high covariation with the right cerebellum and midbrain. These results indicated that both groups recruited similar brain regions, including the putamen and some motor-related regions. However, the functional connectivity was strikingly different between the two groups, as we will describe next.

### Connectivity differences in the stenographer's brain

Complicated cognitive-motor skills, which are typically not automatic, might have become automatic in the stenographers' brain. The cerebellum and putamen (basal ganglia) are typically considered segregated modules in some aspects of learning. The cerebellum is thought to be involved in the adaptive modification of behavior and error-based learning. The basal ganglia, in contrast, is thought to be involved in reward prediction and reward-based learning (Doya 2000; Houk 2005), and in predictions in some types of expertise (shogi, i.e., Japanese chess (Wan et al. 2011)). In the classical view, a distinctive function of cerebellar connections with the cerebral cortex is to gather information from cerebral regions to control movements (e.g., from the primary motor cortex (Allen and Tsukahara 1974)). Such a traditional view may be more consistent with the connections of the control group than of the stenographers. Only the stenographers' training require extensive coordination of linguistic, cognitive, and visual skills under a severe speed constraint, and this may underlie the functional modifications and morphological changes around the striatum. Thus, the coordination of skills for professional stenography appeared to be supported by the striatum, where the lifelong training modulated the various functional modules, including auditory, linguistic, cognitive, mnemonic, sensorimotor, and, possibly, attention, top-down executive, and reward-based learning modules. The questionnaires revealed that the expert stenographers' stenographical performances were mostly automatic. While they pay attention in order to follow the semantic aspects of the speech, every now and then, their attention comes back to checking spelling errors only when necessary. Such introspective observations are highly consistent with other cases of extreme performance expertise (e.g., sports, music, and games) and automatic sensorimotor performances in daily life (e.g., walking, bike riding, and driving a car). Indeed, the activation of the basal ganglia (striatum) is believed to partly reflect automatic motor processing (Jueptner et al. 1997; Floyer-Lea and Matthews 2004; Lehéricy et al. 2005; Poldrack et al. 2005; Jankowski et al. 2009; Reithler et al. 2010; Steele and Penhune 2010) and, at the same time, implicates extreme attention, which is called top-down processing or goal-driven attention (Posner and Petersen 1990; Posner and Rothbart 1998). The career-dependent decrease in supramarginal activity may imply effective performance, which region was involved with language perception and processing (Gazzaniga et al. 2009). Because speech cognition is the first step in dictation, we would be able to examine the career-related effective operation of the following steps of dictation in the expert brain if we can recruit a wider range of ages in this group.

### Comparisons with other types of expert brain

Neural plasticity that is related to various types of expertise has been reported in sports, music, and other fields (For a review, see Chang 2014). A number of studies have revealed that long-term skill learning and repetitive training result in functional and structural changes that enable the expert skills. Cross-sectional studies of elite judoka (Judo wrestler), golfers, and musicians have demonstrated structural changes mostly in the motor- and cognitive-associated regions. For example, elite judo wrestlers have significantly higher volumes of gray matter in several brain regions that are related to motor planning, execution, and working memory (Jacini et al. 2009). Skilled golf players have larger gray matter volumes in the frontoparietal network (Jäncke et al. 2009). Professional musicians exhibit structural differences in the hand motor area of the precentral gyrus compared to nonmusicians (Amunts et al. 1997). Similarly, functional modulations in the expert brain have also been reported. Previous findings have shown more focused activation of the motor-related areas. Professional racquetball players exhibit an enlarged cortical representation of the hand compared to novices (Pearce et al. 2000). Most imaging studies on long-term learning have reported selective modulations of specific brain regions, such as the hippocampus, the precentral gyrus and so on, underlying the specific skills. In our findings, the stenographers' brain exhibited functional and structural modulations in the striatum regions involved in motor control. However, unlike the previous studies, the current findings revealed vigorous plastic changes in both functional and structural components in the stenographers' brain. From this perspective, the current study provided considerable insights into the field of multimodal plasticity in the expert brain.

In summary, the results of the current study were partly consistent with those of previous studies on sensorimotor plasticity, although our results challenge the existing view of expertise by examining the plastic reorganization of the professional expert brain, which is specialized for more complex and integrated skills.

## Supporting Information

**Figure S1.** The left putamen had a specific function in stenographers.

**Figure S2.** Group comparison of the covariated regions of the subregions of the putamen.

**Figure S3.** The subregion specific covariation of the anterior and posterior putamen (comparison analysis against Fig. 5).
