# Supplementary figures and images for: Functional connectivity of the striatum in experts of stenography
Source: Brain Behav. 2015 Mar 25;5(5):e00333. doi: 10.1002/brb3.333 (PMC4396401; doi:10.1002/brb3.333)

A) Writing

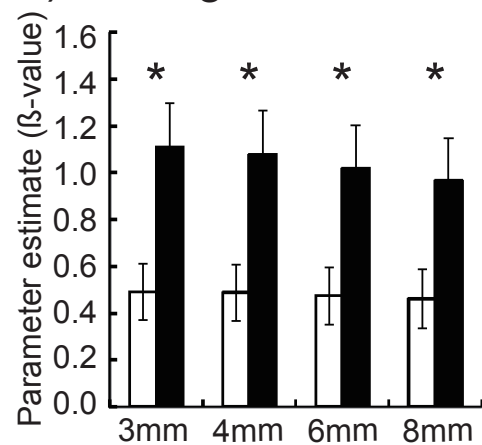

B) Imaging

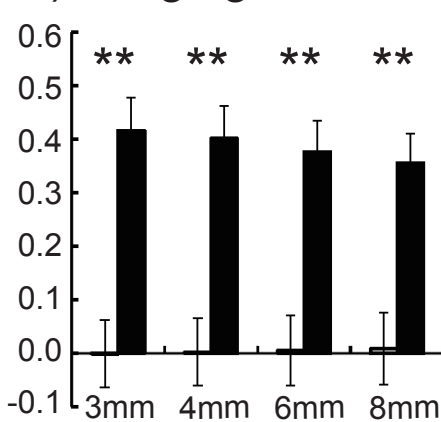

C) Hearing

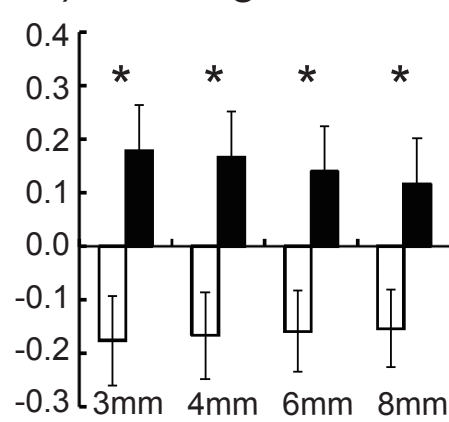

Supplement: Supplementary file 1 — Figure S1. The left putamen had a specific function in stenographers. [file brb30005-e00333-sd1.pdf]

# A) Anterior Putamen (Stenogarpher > Control)

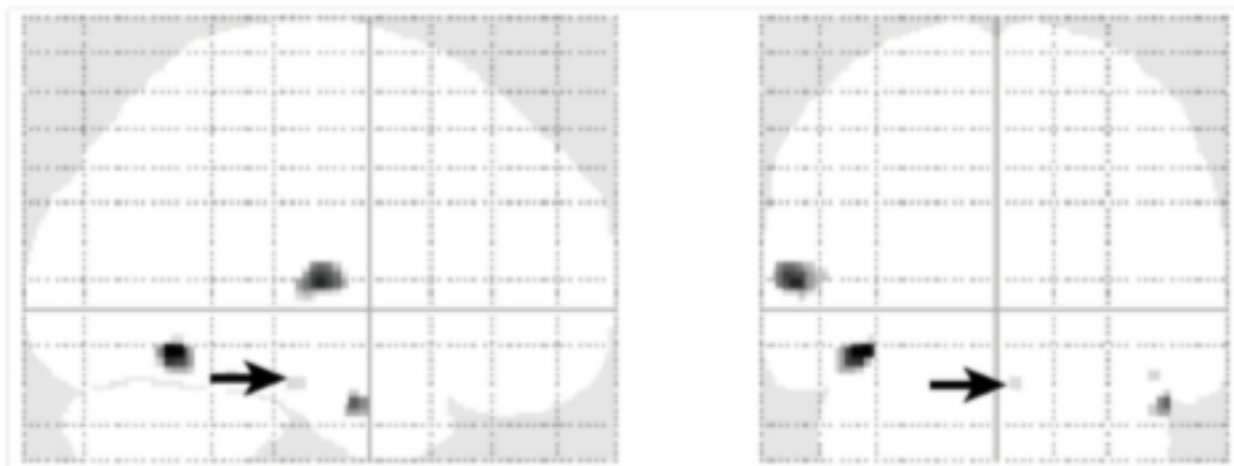

# B) Posterior Putamen (Control > Stenogarpher)

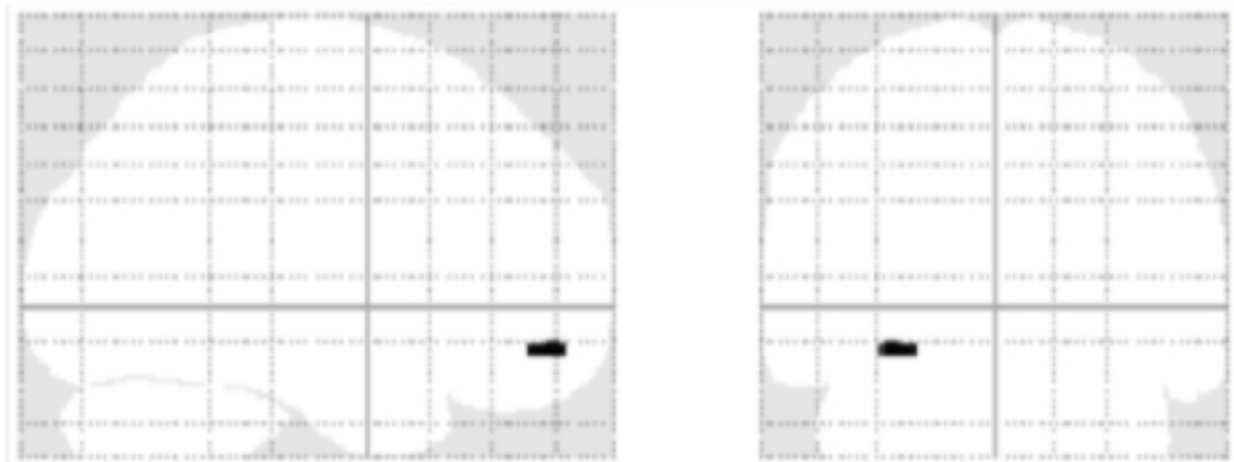

Supplement: Supplementary file 2 — Figure S2. Group comparison of the covariated regions of the subregions of the putamen. [file brb30005-e00333-sd2.pdf]

# A) Stenographer

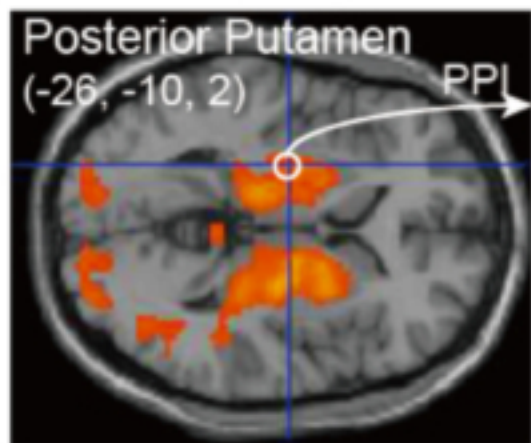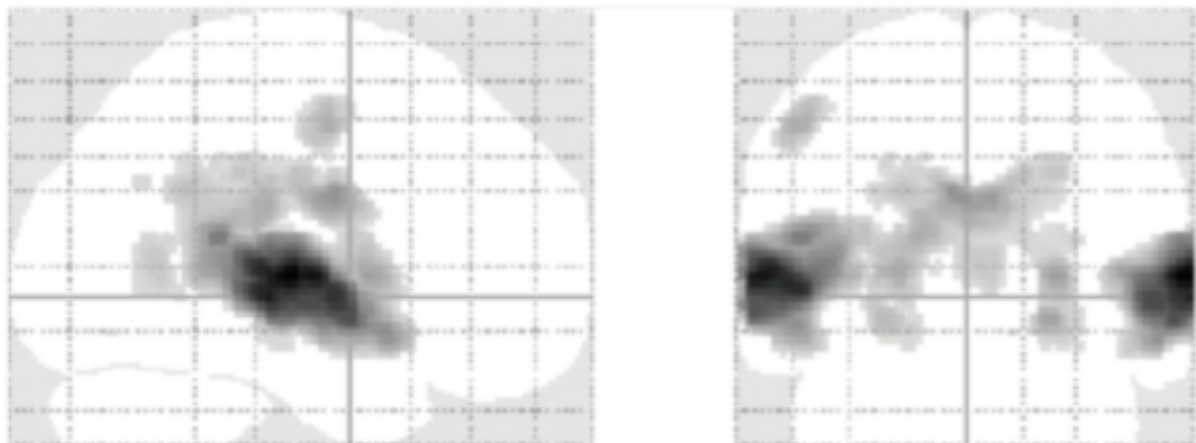

# B) Control

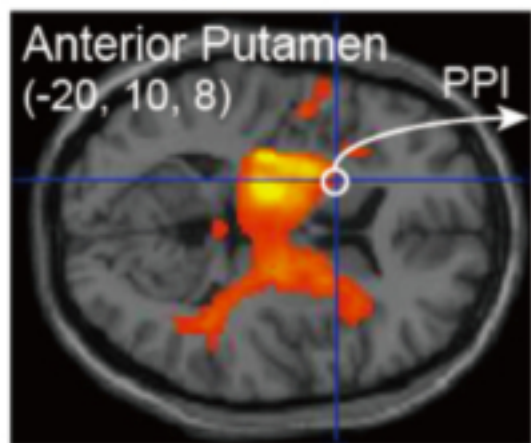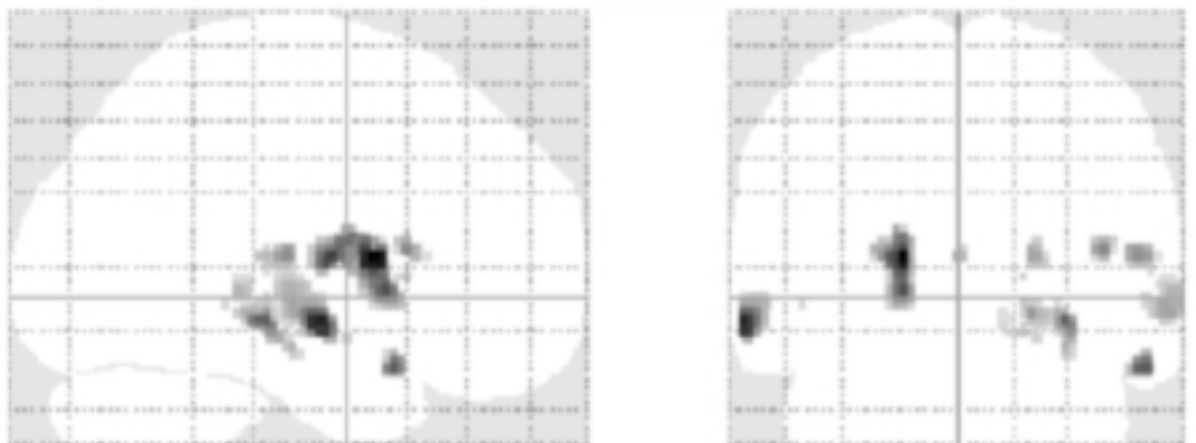

Supplement: Supplementary file 3 — Figure S3. The subregion specific covariation of the anterior and posterior putamen (comparison analysis against Fig. 5). [file brb30005-e00333-sd3.pdf]
