# Supplementary material for: Functional connectivity of the striatum in experts of stenography
Source: Brain Behav. 2015 Mar 25;5(5):e00333. doi: 10.1002/brb3.333 (PMC4396401; doi:10.1002/brb3.333)
Supplement: Supplementary file 4 [file brb30005-e00333-sd4.docx]

**Supplementary Figure S1. The left putamen had a specific function in stenographers.**

To examine the objectivity of the ROI analysis, we extracted the spherical ROIs with the several radii. Regardless of the conditions (Writing (*A*), Imagining (*B*) and Hearing (*C*)) and the radius of spherical ROIs, the left putamen of the stenographers exhibited higher activity than controls.　The consistent results with various radii of the spherical ROIs indicated that the anterior regions the left putamen was the specific core region of the stenographic motor skills. Each bar graph indicated averaged ß-values of each ROI and the black bars and white bars indicated the stenographers and the controls, respectively. The horizontal axis indicated the radius of spherical ROIs. The centers of ROI were defined by the peak voxels of the stenographers vs. controls contrast (Fig. 1*A - C*). The value of bar graphs meant the average, and the error bars indicated S.E. ** *p* < .001, * *p* < .05.

**Supplementary Figure S2. Group comparison of the covariated regions of the subregions of the putamen.** The group comparison of the PPI analysis of the anterior (*A*) and the posterior (*B*) putamen. The stenographer showed a higher covariated region in the midbrain (4, -24, -22; arrow in (*A*)) , but there were no statistical differences around the cerebellum and the midbrain in other conditions. For the PPI analysis of the anterior putamen, we used the coordinate (-20, 10, 8), because of the same reason of Supplementary Fig. S . On one hand, for the posterior region, the coordinate (-26, -10, 2) was used for the stenographers, and the coordinate (-30, -18, 6) was for the controls, which were the peak voxels for each group. The statistical threshold was *p* < .001 (uncorrected).

**Supplementary Figure S3. The subregion specific covariation of the anterior and posterior putamen (comparison analysis against Figure 5).** The left panel of (*A*) showed the peak voxels (white circles) in the posterior putamen of the stenographers, which was also used as the seed regions for PPI analysis. The left panel of (*B*) showed the activation of the anterior putamen of the control, but there were not a peak voxel in this subregion, so we used the coordinate (-20, 10, 8 (*p*<.001 (uncorrected)), which is the same coordinate to use the PPI analysis for the anterior putamen of the stenographer. There were no covariated regions around the cerebellum and the midbrain in both groups. An uncorrected threshold *p* < .0001 was applied to the left panels of (*A*) and (*B*), and *p* < .001 (uncorrected) was applied to the middle and right panels of (*A*) and (*B*). Black arrows indicate the peak voxels.
